# eIF2α Kinases Regulate Development through the BzpR Transcription Factor in *Dictyostelium discoideum*

**DOI:** 10.1371/journal.pone.0032500

**Published:** 2012-03-05

**Authors:** Charles K. Singleton, Yanhua Xiong, Janet H. Kirsten, Kelsey P. Pendleton

**Affiliations:** Department of Biological Sciences, Vanderbilt University, Nashville, Tennessee, United States of America; University of Dundee, United Kingdom

## Abstract

**Background:**

A major mechanism of translational regulation in response to a variety of stresses is mediated by phosphorylation of eIF2α to reduce delivery of initiator tRNAs to scanning ribosomes. For some mRNAs, often encoding a bZIP transcription factor, eIF2α phosphorylation leads to enhanced translation due to delayed reinitiation at upstream open reading frames. *Dictyostelium* cells possess at least three eIF2α kinases that regulate various portions of the starvation-induced developmental program. Cells possessing an eIF2α that cannot be phosphorylated (BS167) show abnormalities in growth and development. We sought to identify a bZIP protein in *Dictyostelium* whose production is controlled by the eIF2α regulatory system.

**Principal Findings:**

Cells disrupted in the *bzpR* gene had similar developmental defects as BS167 cells, including small entities, stalk defects, and reduced spore viability. β-galactosidase production was used to examine translation from mRNA containing the *bzpR* 5′ UTR. While protein production was readily apparent and regulated temporally and spatially in wild type cells, essentially no β-galactosidase was produced in developing BS167 cells even though the *lacZ* mRNA levels were the same as those in wild type cells. Also, no protein production was observed in strains lacking IfkA or IfkB eIF2α kinases. GFP fusions, with appropriate internal controls, were used to directly demonstrate that the *bzpR* 5′ UTR, possessing 7 uORFs, suppressed translation by 12 fold. Suppression occurred even when all but one uORF was deleted, and translational suppression was removed when the ATG of the single uORF was mutated.

**Conclusions:**

The findings indicate that BzpR regulates aspects of the development program in *Dictyostelium*, serving as a downstream effector of eIF2α phosphorylation. Its production is temporally and spatially regulated by eIF2α phosphorylation by IfkA and IfkB and through the use of uORFs within the *bzpR* 5′ UTR.

## Introduction

One mechanism of translational control of gene expression utilizes the translational initiation factor eIF2. This multi-subunit protein, in what is referred to as the ternary complex, delivers initiator tRNAs to scanning ribosomes assembling on mRNAs. Phosphorylation of the subunit eIF2α at serine 51 reduces the ability to deliver initiator tRNAs and thus the efficiency of translation [1]. Upon phosphorylation of eIF2α overall translation generally decreases. However, the translation of a small number of specific mRNAs actually can be enhanced in the presence of phosphorylated eIF2α [2]–[7] by a mechanism using short upstream open reading frames (uORFs) within the 5′ untranslated regions (UTRs) of these mRNAs [8]. There are four different types of eIF2α specific kinases: GCN2, PERK, HRI, and PKR. These kinases are activated by various cellular and environmental stimuli through regulatory domains that control the activity of the kinase domain [1]. The conditions that activate the kinases and trigger eIF2α phosphorylation often are various stresses that are survived or overcome by activating the eIF2 regulatory system.

Many of the mechanistic insights of the eIF2 regulatory system and eIF2α specific kinases came from studies of the yeast GCN2 protein which, when activated by stresses such as general nutrient limitation and/or amino acid starvation, subsequently activates GCN4 translation [1], [8]. Under low levels of phosphorylated eIF2α, ribosomes scan the 5′ UTR, translate an initial uORF, and are able to reinitiate at one or more of several subsequent uORFs due to efficient delivery of the ternary complex. The ribosomes are released after the reinitiation without further scanning, thus resulting in little or no translation of the true coding region that lies downstream of the uORFs. High levels of phosphorylated eIF2α lead to a low concentration of the ternary complex and thus delayed reinitiation. Delivery to the scanning ribosome occurs after the ribosome has passed the uORFs but prior to reaching the true coding region, resulting in engagement and translation of the encoded protein [3], [9].

Proteins that are encoded by mRNAs whose translation is upregulated upon eIF2α phosphorylation often are transcription factors within the basic leucine zipper (bZIP) family [3], [5], [7], [10], [11]. Enhanced translation of these proteins results in the transcription of a number of genes whose protein products assist the cell in overcoming the stress that triggered the increased eIF2α phosphorylation.

*Dictyostelium* cells have three GCN2 homologs that are termed initiation factor kinases: IfkA, IfkB, and IfkC. Three other uncharacterized genes have kinase domains similar to eIF2α kinases but lack obvious regulatory domains. All three of the *ifk* genes are expressed during growth and throughout development, with a high degree of spatially restricted expression for each gene during development [12], [13]. Disruptions of the *ifk* genes indicate that IfkA functions in modulating the timing of aggregation and early gene expression, that IfkB functions in maintaining proper prestalk specific gene expression, and that IfkA and IfkB function in maintaining proper cell-cell and cell-substrate adhesion and the equilibrium between different cell types for proper spatial patterning [12], [13]. eIF2α phosphorylation recently was shown to negatively affect *Dictyostelium* cell proliferation via direct regulation of two chalone proteins [14].

In the latter study, a strain was used (BS167) that possesses an eIF2α that cannot be phosphorylated due to an S51A mutation. This strain shows several developmental defects, including smaller size of the developing entities, various stalk defects, and production of non-viable spores. We wondered if one or more of these defects were the result of the lack of production of a bZIP transcription factor, given the inability to phosphorylate eIF2α in BS167. *Dictyostelium* possesses 19 potential bZIP family members [15], and the work presented herein focuses on one of these, namely BzpR. We demonstrate that BzpR production is translationally regulated during development via eIF2α phosphorylation by IfkA and IfkB and through the use of uORFs within the *bzpR* 5′ UTR.

## Materials and Methods

### Disruption of *bzpR*

A disruption construct, pbzpR-5, was made as follows. The blasticidin resistance gene cassette from pBSR519 [16] was inserted into a BamHI site between a 500 bp 5′ region of the *bzpR* gene (covering the 5′ UTR and the first few codons) and a 900 bp 3′ region (corresponding to codons 370 to 670) that had been cloned into the pGEM T-easy vector (Promega). The 5′ fragment was generated by PCR using primers bzr-3 and bzr-10, while the 3′ fragment was made with bzr-7 and bzr-8 (Table 1). Digestion with BstXI and EcoRI was carried out to release the bzpR/bsr fusion prior to transformation into *Dictyostelium* cells via electroporation. To check for disruption, genomic DNA was isolated from blasticidin resistant clones and used as a template in a PCR reaction with a blasticidin specific primer and a *bzpR* primer downstream of the cloned 3′ region. Several independent isolates produced strains with the same phenotype and one (BS168) was used for the experiments in this paper.

**Table 1:** Oligonucleotides used in this study.

Oligo	Sequence
bzr-3	CTGGCGCTAACAAAGGATTTTC
bzr-5	ATATATTCCCTCTTGCAATCAT
bzr-7	ggatccCAACAATTACAAGAGCAACAGG
bzr-8	CAAGTAATGAATATCTTTGATC
bzr-9	CATTATTAAAACATTCTAATCTGACAC
bzr-10	GATCTATTTATCCATACTAAATTC
bzr-12	tctagACCATTCTCTGGCCCAGTTCA
bzr-13	agatctTGGCGCTAACAAAGGATTTTC
bzr-16	gatctaagcttAAAAATGGATAATTTTGAAAATCCTTTGTTAGCGA
bzr-17	ctagtCGCTAACAAAGGATTTTCAAAATTATCCATTTTTaagctta
bzr-18	actagtCGCTAACAAAGGATTTTCAAAATTATC
bzr-19	agatcTATATATTCCCTCTTGCAATCAT
bzr-20	ATTTTATTCAATTTTAATAtg
H7Q1	ATTAGGTGGTGCCAATC
H7Q2	GTGGGCTCTTAATTGAAC
GFP-5	CACTGGAGTTGTCCCAATTC
GFP-3	GTCTGCCATGATGTATACAT
RFP-5	ATTGAAGGTGAAGGTGAAGG
RFP-3	GCACCTGTTGAATGTCTACC
lacZ-4	CACGACGTTGTAAAACGACG

Upper case sequence occurs in genomic DNA.

### Generation of 5′ UTR GFP and RFP fusions

**Figure 1:**
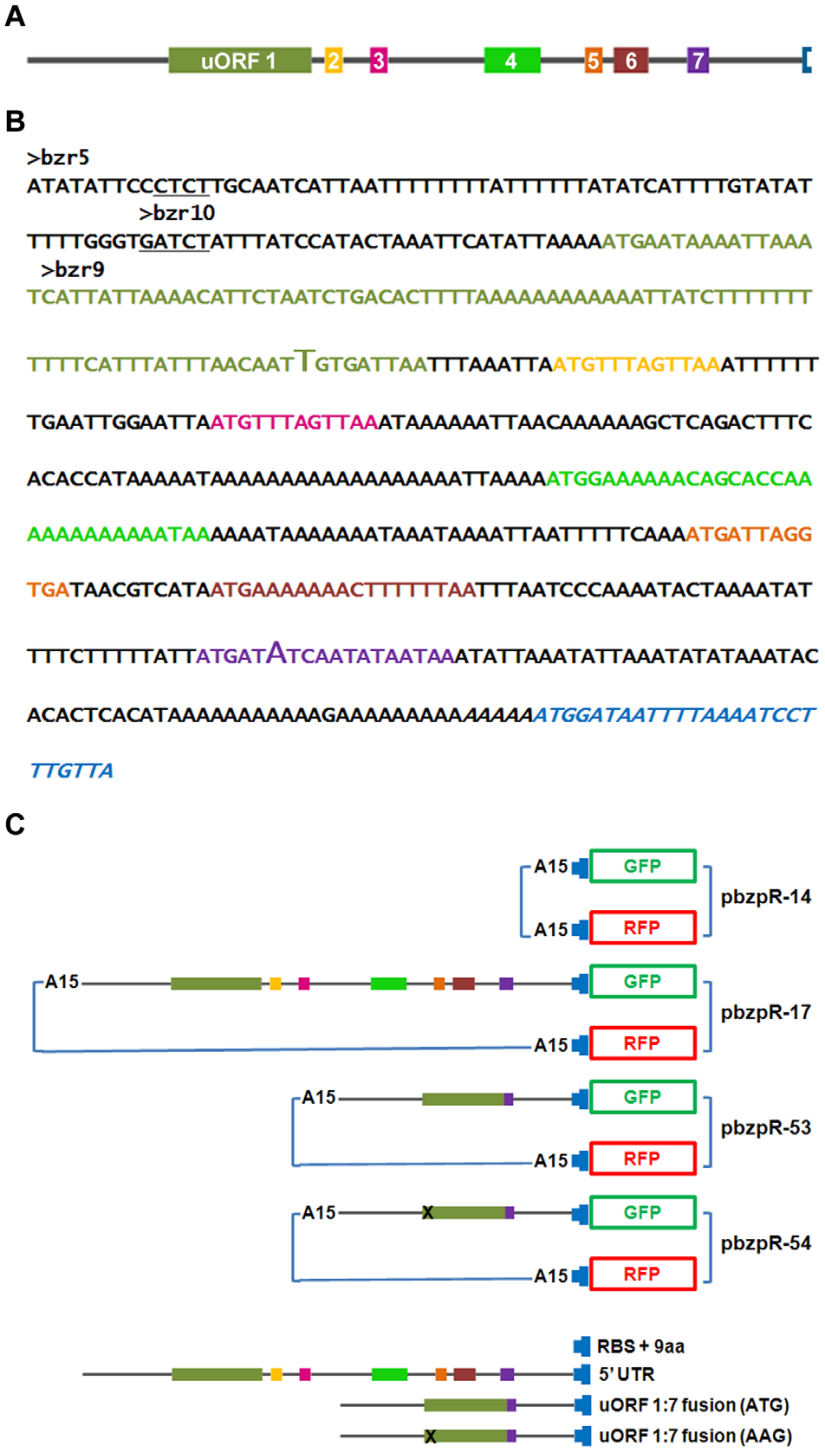
The *bzpR* 5′ UTR sequence and plasmids used to test translational suppression. **A**. A ‘to scale’ schematic of the seven uORFs within the *bzpR* 5′ UTR, color coded to correspond with panels B and C. **B**. The *bzpR* 5′ UTR sequence and the first nine codons (blue) of the coding region. The 5′ ends of the three primers, bzr-9, bzr-10, and bzr-5, used in RT-PCR to localize the transcriptional start site, are indicated. Transcription likely starts between the two underlined regions. The sequence shown was fused to the GFP gene in pbzpR-17. The italicized region, consisting only of the ribosome binding site and the first 9 codons, is the no-UTR control fused to the GFP and RFP genes in pbzpR-14 and to the RFP gene in pbzpR-17, -53, and -54. The larger font T and A in the first and last uORFs represent the fusion junction in the internal deletion constructs (pbzpR-53 and -54). **C**. Schematic diagram of the plasmids used to demonstrate suppression of translation by the *bzpR* 5′ UTR. A15 represents the actin 15 promoter driving transcription of each fusion. Thin blue lines represent the remaining portions of the plasmids, including transcriptional terminators and selectable drug resistance genes.

A *bzpR* no-UTR control sequence was generated by annealing the bzr-16 and bzr-17 oligonucleotides (Table 1) and cloning the resulting hybrid into the BglII and SpeI sites of pDM323 for fusion to GFP and into pDM330 for fusion to RFP [17]. The annealed oligonucleotides possessed BglII and SpeI sticky ends, the ribosome binding site, and first 9 amino acids of *bzpR* (Fig. 1). The expression cassette from the RFP fusion, possessing the actin 15 promoter, the *bzpR* no-UTR, the RFP gene, and the actin 8 terminator, was released by digestion with NgomIV and cloned into the NgomIV site of the no-UTR/GFP fusion plasmid to give pbzpR-14. This served as the control plasmid with the GFP and RFP genes both under the control of the actin 15 promoter (transcription) and the *bzpR* no-UTR (translation) (Fig. 1C).

The 540 bp *bzpR* 5′ UTR and first 9 amino acid codons (Fig. 1B) were amplified using primers bzr-18 and bzr-19 (Table 1), cloned into the pGEM T-easy vector, and sequenced. The fragment was released by digestion with BglII and SpeI and cloned into pDM323 digested with the same enzymes. Following digestion of the resulting plasmid with NgomIV, the no-UTR RFP expression cassette described above was inserted. The resulting plasmid, pbzpR-17, possesses the GFP gene controlled by the actin 15 promoter (transcription) and the *bzpR* 5′ UTR (translation) while the RFP gene is controlled by the actin 15 promoter and the *bzpR* no-UTR (Fig. 1C).

A mutated version of pbzpR-17 with the first and seventh uORFs fused and all other uORFs deleted (Fig. 1) was made by digesting the *bzpR* 5′ UTR with MfeI, filling in with Klenow polymerase, digesting with EcoRV, and ligating the 5′ and 3′ pieces. This internally deleted 5′ UTR was fused as above to the GFP gene and the no-UTR RFP cassette was cloned into this plasmid to give pbzpR-53 (Fig. 1C). These steps also were carried out on a version of the 5′ UTR for which the ATG of the first uORF was mutated to AAG to give pbzpR-54 that is identical to pbzpR-53 except for the mutation of the uORF start codon. The mutation was generated as described [18] using the “mutant” oligonucleotide bzr-20 (Table 1).

### Fusing the *bzpR* promoter and 5′ UTR to *lacZ*

PCR using bzr-12 and bzr-13 (Table 1) and genomic DNA gave a 1960 bp fragment corresponding to the 5′ upstream sequences and the start codon of the *bzpR* gene. After sequencing, the fragment was used to replace the *ecmAO* promoter in pEcmAO-i-α-gal (BglII and XbaI digestion) and thus become fused to a rapid turnover version of β-galactosidase. The resulting plasmid was named pbzpR-9.

### Cell growth and development

Strains were maintained and grown in HL-5 medium [19] at 21°C. For development, cells were grown in the presence of *Klebsiella pneumoniae* on SM plates, harvested, and excess bacteria removed by differential centrifugation prior to plating cells on nitrocellulose filters for standard development [20]. Staining for β-galactosidase activity was carried out as described [21].

### RT-PCR

RNA was isolated using Trizol (Invitrogen). RT-PCR was carried out as described [22] with the inclusion of a DNase treatment step to insure removal of genomic DNA. Various primer pairs were used as listed in the results section and shown in Table 1. For each primer pair, RNA concentrations, annealing temperatures, and cycle numbers were optimized to maximize sensitivity to variations in RNA levels between samples. In each case, differences in mRNA levels of two to ten-fold were readily detected. Controls with no reverse transcriptase were included to demonstrate no genomic DNA contamination existed. Oligonucleotides specific for the H7 gene were used as an internal control as a constitutively transcribed gene during growth and development [23].

### GFP and RFP fluorescence quantitation

Strains transformed with the 5′ UTR GFP/RFP fusion plasmids and strains with GFP and RFP expressed individually were maintained in HL-5 plus 10 µg/ml G418 or 50 µg/ml hygromycin. Prior to their use, the strains and the parental wild type Ax4 strain were grown for 72 hours in Loflo+ medium (http://www.dictybase.org/techniques/media/lowflo_medium.html) under appropriate selection to minimize cell auto-fluorescence. To minimize media fluorescence, the cells were transferred to fresh Loflo+ medium the night before the experiment and transferred to Loflo medium, which lacks yeast extract, six hours prior to the experiment. The cells were pelleted and suspended in fresh Loflo at a titer of 2×10^6^ immediately prior to flow cytometry.

Flow cytometry was conducted on a 5-laser BD LSR Fortessa Special Order Research Product running BD FACSDiva Software Version 6.1.3. Prior to collecting data, the cytometer was calibrated using the non-fluorescing Ax4 strain and the strains individually transformed with either pRFP or pGFP as controls. Five thousand events were collected for the controls and approximately 12,000 events were collected for each experimental strain. The resulting FCS files were analyzed with Cytobank (www.cytobank.org) using the control data for compensation and gating.

### Spore viability analysis

Spore filled sori from Ax4 and BS168 cells growing on bacteria were picked and suspended in PDF (22 mM potassium phosphate, pH 6.5, 20 mM KCl, 5 mM Mg_2_Cl). After two washes in PDF, spores were titered and suspended in 10 mM EDTA, pH 7.5, 0.1% NP40. The samples were incubated at 42°C for 45 minutes followed by two washes with PDF. Spores were suspended with *Klebsiella pneumoniae* and were plated at 100 spores per plate onto SM plates [24]. Colonies, derived from individual spores, were counted after incubation for several days at 21°C. Three independent experiments were performed.

### Microscopy and image processing

Developing cells and β-galactosidase results were photographed with a Leica MZ16 stereomicroscope equipped with a Q-Imaging Retiga 1300 camera and Q-Imaging software. Images were oriented and cropped using Adobe Photoshop CS 3, version 10.0.1.

## Results

### Defining the 5′ UTR of the *bzpR* gene

The upstream regions of the 19 *bzp* genes of *Dictyostelium* were searched for upstream open reading frames (uORFs). While several of the genes possess uORFs, only a few had distributions somewhat analogous to that of GCN4 of yeast, the prototypical gene regulated by eIF2α phosphorylation [1]. The work herein focuses on one such *Dictyostelium* gene, *bzpR*, whose 5′ upstream sequence is shown in Figure 1. There are 7 uORFs within the first 540 bp upstream of the start codon of *bzpR*. Typically for *Dictyostelium* genes, transcription start sites are located much closer to the ATG of the coding region. Thus, we determined if these uORFs were present within the *bzpR* mRNA in what would be a lengthy 5′ UTR.

**Figure 2:**
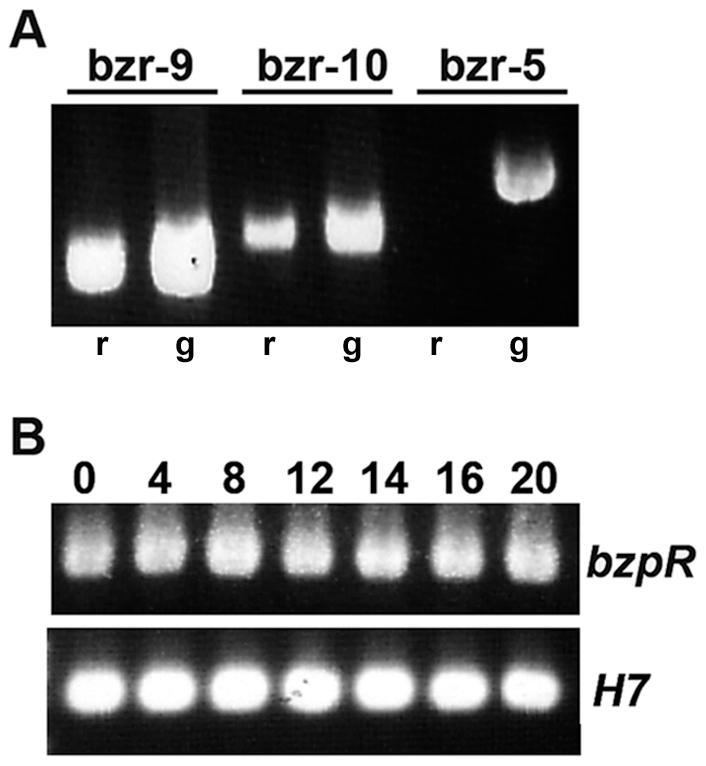
RT-PCR localization of transcriptional start site of *bzpR* gene and determination of relative mRNA levels. **A**. RNA and genomic DNA were isolated from growing Ax4 cells and used as templates in RT-PCR (r) or PCR (g) reactions. A 3′ primer (bzr-3) corresponding to sequences at the beginning of the coding region was used with three different 5′ primers corresponding to sequences progressively further upstream of the coding region (see Fig. 1A). Bzr-9 lies within the first uORF, bzr-10 lies just upstream of uORF 1, and bzr-5 is 41 residues further upstream of -10. **B**. RNA was isolated from Ax4 growing cells (0) and cells plated for development for the indicated times (in hours) and used in RT-PCR reactions with bzr-3 and -10. *H7* specific primers were used as an internal control as *H7* is expressed constitutively during growth and development. Conditions were optimized to reveal differences in RNA levels of two to ten-fold.

Oligonucleotides corresponding to various positions within the upstream sequence, paired with a 3′ primer just within the coding region, were used in RT-PCR reactions on RNA and PCR reactions on genomic DNA. The results using three such primers are shown in Figure 2A, with the position of the 5′ end of each primer in relation to the uORFs shown in Figure 1B. Both primers bzr-9 and bzr-10 gave expected size products using either RNA or genomic DNA as template. The bzr-9 primer lies within the first (5′ most distal) uORF while bzr-10 is just upstream of the first uORF. In contrast, a third primer, bzr-5, that lies 41 residues upstream of bzr-10 gave the expected size band with genomic DNA as template but gave no band in the RT-PCR reaction. These results indicate that the *bzpR* mRNA has a lengthy 5′ UTR covering all of the 7 uORFs, with the transcriptional start site most likely located 30 to 50 nucleotides upstream of uORF 1.

Figure 2B shows the results of RT-PCR assaying for *bzpR* mRNA presence during growth and development. Expression of *bzpR* mRNA was found during growth and throughout the developmental program, with some increase in levels from 8 to 12 hours evident in some experiments. Recent RNA-seq data give a similar pattern but show a more pronounced increase around 8 hours (http://dictyexpress.biolab.si/).

### *BzpR* null cells show developmental defects

**Figure 3:**
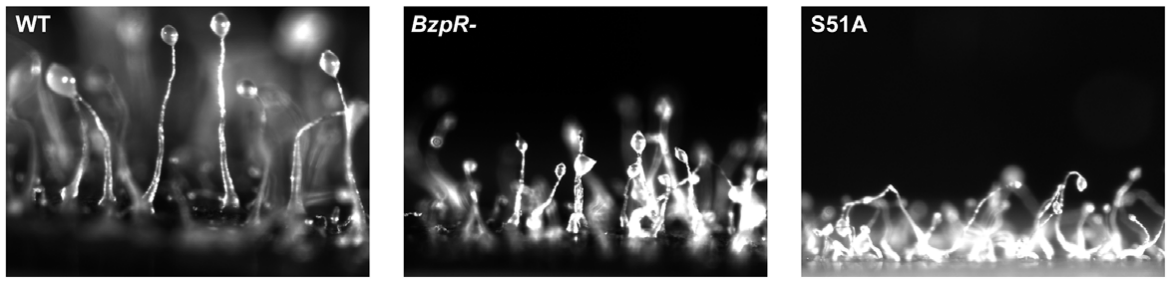
Fruiting bodies of Ax4 (wild type), BS168 (*bzpR* null), and BS167 (eIF2α S51A knockin). Cells were grown in the presence of bacteria, harvested, and plated for development. Photographs were taken at 48× after the filter was adhered to a gel slice in a vertical position.

Disruption of the *bzpR* gene was carried out, and the null cells (BS168) were developed to compare their phenotype to that of wild type cells (Ax4). The developing entities were small (Fig. 3), an aberration evident early on at the mound stage. In addition, the fruits that formed had various stalk deficiencies, with the most obvious being a thicker than normal lower third of the stalk and “droopy” sori that angle downward instead of being supported upright at the anterior ends of the stalks. Interestingly, similar developmental aberrations are seen in BS167 (Fig. 3). BS167 possesses a mutated eIF2α gene (S51A mutation) that results in an eIF2α protein that cannot be phosphorylated [14]. Previous work showed that spores of BS167 are mostly nonviable, being only 7% of wild type viability [14]. Similarly, we found that *bzpR* null cells produced spores with only 17% of wild type viability. These similarities in developmental defects suggest that BzpR may be a downstream effector of eIF2α phosphorylation.

### Proper BzpR protein production requires eIF2α phosphorylation

**Figure 4:**
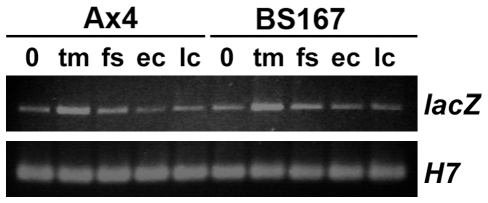
RT-PCR determination of relative levels of β-galactosidase mRNA in Ax4 and BS167 transformed with pbzpR-9. Wild type (Ax4) and eIF2α S51A knockin (BS167) cells were grown in the presence of bacteria, harvested, and plated for development. RNA was isolated from growing cells (0) and at the tipped mound (tm), finger/slug (fs), early culminant (ec), and late culminant (lc) stages. Primers used were bzr-10 and lacZ-4. H7 primers were used in parallel reactions to confirm RNA concentrations. Conditions were optimized to reveal differences in RNA levels of two to ten-fold.

To examine the spatial and temporal expression of the BzpR protein and gain insight into whether its production depends on eIF2α phosphorylation, a 1960 bp fragment, corresponding to the upstream sequences (promoter and 5′ UTR) and the start codon of *bzpR*, was fused to the β-galactosidase gene as an easily detectable surrogate for the BzpR protein. The resulting plasmid was transformed into Ax4 and BS167 (eIF2α S51A knockin) cells, transformants were plated for development, and the *lacZ* mRNA and protein produced in each strain was compared. The same pattern and importantly the same levels of *lacZ* mRNA were produced in both the Ax4 and BS167 strains (Fig. 4).

**Figure 5:**
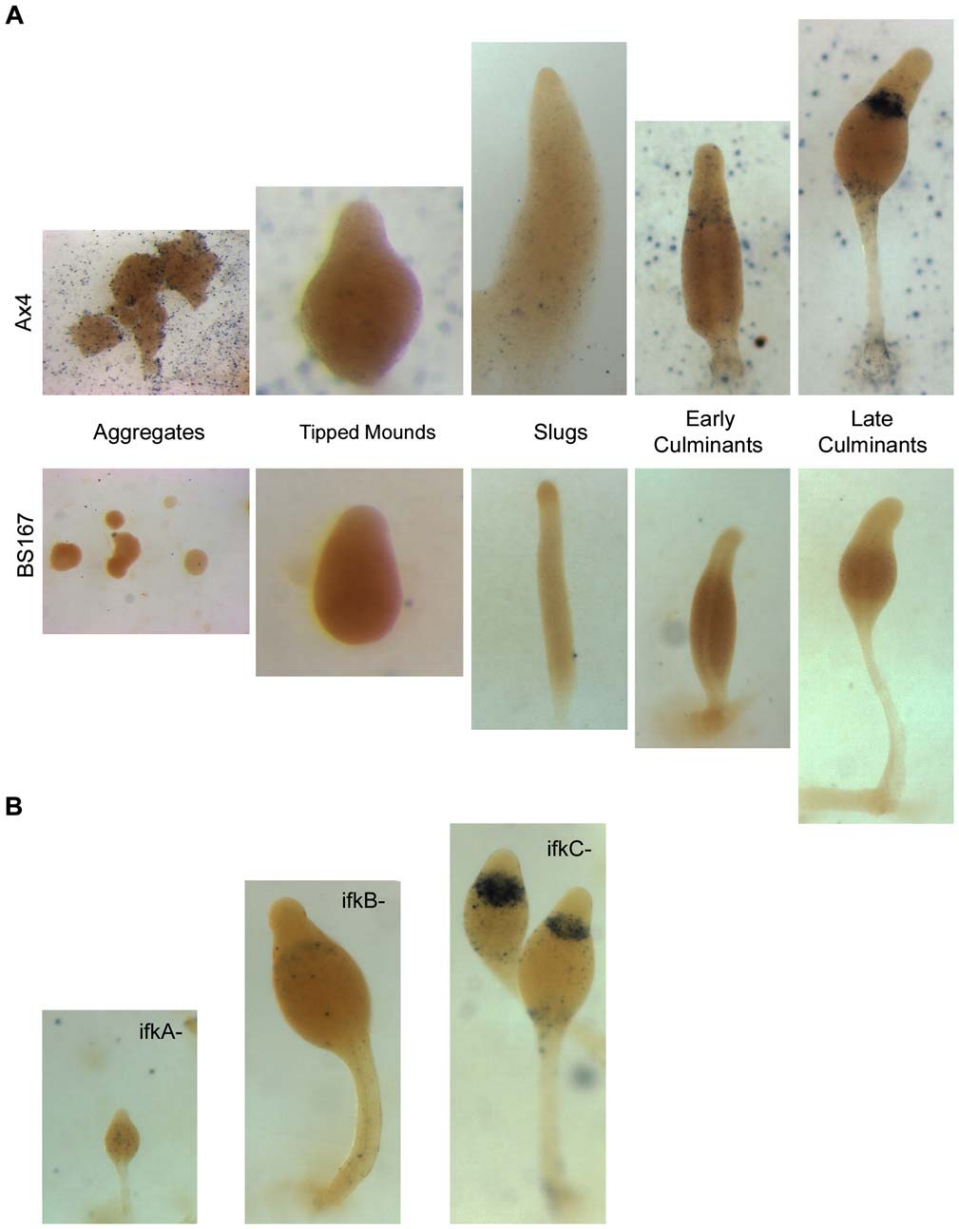
Spatial and temporal expression of β-galactosidase protein driven by the *bzpR* promoter and 5′ UTR. Strains transformed with pbzpR-9 were grown in the presence of bacteria, harvested, and plated for development. At appropriate times, filters of the developing cells were fixed and stained for β-galactosidase activity. All samples were stained for 5 hours at room temperature, washed, and photographed in glycerol at 48× with the exception of tipped mounds, which were photographed at 75× to capture detail. **A**. Various stages are shown for Ax4 (wild type) and BS167 (eIF2α S51A knockin). **B**. Late culminants are shown for BS153 (*ifkA* null), BS160 (*ifkB* null), and BS166 (*ifkC* null).

In Ax4 cells the production of the LacZ protein varied temporally and spatially, but in striking contrast little to no protein was observed in BS167 cells (Fig. 5). For the wild type Ax4 strain, a small number of cells scattered throughout the cell population expressed β-galactosidase prior to aggregation. A number of these cells did not enter the aggregating entities, but many did as seen by the speckled staining in aggregates (Fig. 5A). The intensity of speckling and the number of speckles decreased as tipped mounds formed, even though this was the time of peak mRNA levels. Tipped mounds, first fingers, and slugs showed faint speckled staining throughout the entities, with staining in aged slugs confined to the prespore region. As culmination began, staining intensity and numbers of speckles increased, especially at the boundary between prespore and prestalk cells (upper cup cells). By mid to late culmination, intense upper cup staining was found, and subsequently upper cup staining usually decreased as the tipped fruits matured. In striking contrast, little to no staining was seen in the eIF2α S51A knockin strain at any stage of development (Fig. 5A), even though *lacZ* mRNA levels were the same as those in Ax4 cells. If the staining process was carried out at 37°C and for extended times, transformed populations of BS167 did show faint scattered speckling during culmination.

Thus, protein production was regulated both temporally and spatially in the wild type strain. Importantly, protein production was dependent on the presence of an eIF2α that can be phosphorylated as revealed by the lack of staining in BS167 cells. A role for eIF2α phosphorylation in producing protein from mRNA containing the *bzpR* 5′ UTR was substantiated by finding minimal staining and no upper cup expression in strains that lacked either IfkA (BS153) or IfkB (BS160) (Fig. 5B). IfkA and IfkB are known eIF2α kinases [12], [13]. In contrast to the findings for the *ifkA* and *ifkB* null strains, β-galactosidase expression was normal in the *ifkC* null strain (BS166), another eIF2α kinase (Fig. 5B).

### Role of uORFs in BzpR production

Regulation of yeast GCN4 protein production by eIF2α phosphorylation requires the presence of uORFs in the GCN4 mRNA that suppress translation in the absence of phosphorylated eIF2α [1]. To determine if the *bzpR* uORFs mediate the translation suppression demonstrated above for the *bzpR* 5′ UTR fused to the *lacZ* gene, either the *bzpR* upstream sequences containing the 7 uORFs plus the first nine codons (5′ UTR) or a sequence corresponding only to the ribosome binding site and the first nine codons (no-UTR) was fused to a GFP gene that was transcriptionally controlled by the actin 15 promoter. As an internal control within both of the resulting plasmids, the ribosome binding site and the first nine codons (no-UTR) was fused to an RFP gene under the control of another copy of the actin 15 promoter [17]. Thus, pbzpR-14 serves as a control with both the GFP and RFP in the no-UTR form, while pbzpR-17 possesses a GFP potentially regulated by the *bzpR* 5′ UTR and a no-UTR RFP (Fig. 1B).

**Figure 6:**
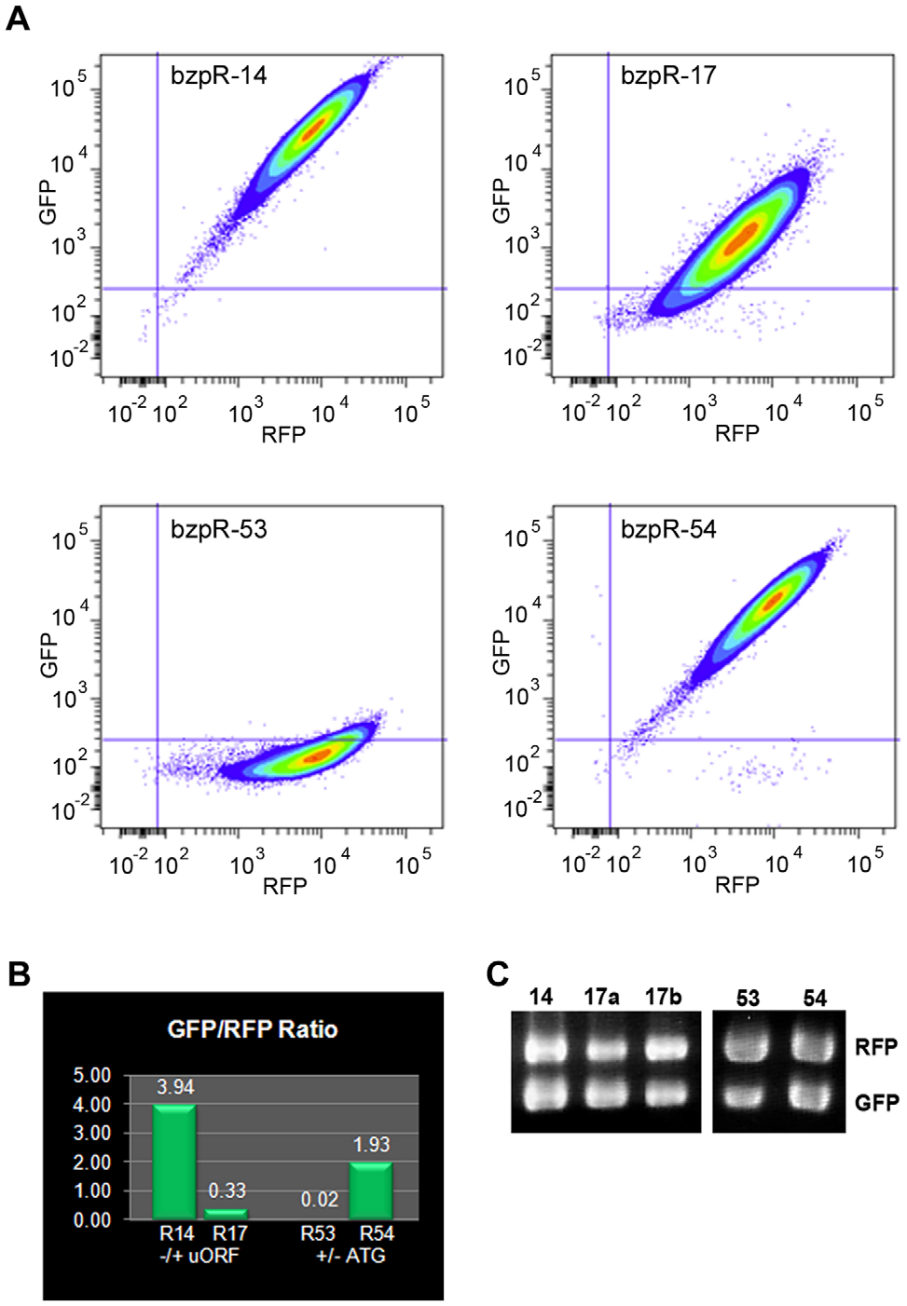
Translational repression by the *bzpR* 5′ UTR. Ax4 cells were transformed with the various *bzpR* 5′ UTR constructs: pbzpR-14 (no-UTR GFP, no-UTR RFP), pbzpR-17 (5′ UTR GFP, no-UTR RFP), pbzpR-53 (GFP: modified 5′ UTR with a single uORF, that being the fusion of uORF 1 and 7; no-UTR RFP), or pbzpR-54 (GFP: modified 5′ UTR mutant with the single fused uORF possessing an AAG start codon mutation; no-UTR RFP). Flow cytometry was used to compare levels of GFP and RFP in cells of each strain. **A**. Colored contour plots of GFP versus RFP fluorescence intensities for cells transformed with the indicated constructs. **B**. The GFP and RFP mean fluorescence values from the contour plots were used to determine the GFP to RFP ratios for each strain. Mean values were: pbzpR-14, 34,372 (GFP), 8735 (RFP); pbzpR-17, 1863 (GFP), 5675 (RFP); pbzR-53, 172 (GFP), 9570 (RFP); pbzpR-54, 19,193 (GFP), 9966 (RFP). **C**. RNA was isolated from cells transformed with each construct, and RT-PCR was carried out with RFP and GFP specific primers to compare relative levels of RFP and GFP mRNA. Two independently transformed populations are shown for pbzpR-17 (a and b). Several independently transformed populations with pbzpR-53 or pbzpR-54 gave identical results to those shown, i.e., a slight reduction in relative levels of GFP mRNA in -53.

Both plasmids were transformed into wild type *Dictyostelium* cells. Flow cytometry was used on populations of transformed cells to examine the relative levels and ratio of GFP to RFP produced by each configuration. If the *bzpR* 5′ UTR suppresses translation, the GFP to RFP ratio for cells possessing pbzpR-17 should be reduced relative to that of pbzpR-14 due to decreased GFP production. This was indeed the case as seen in Figure 6A and 6B, with the *bzpR* 5′ UTR resulting on average in a 12 fold reduction in GFP production relative to the no-UTR control. The reduced GFP to RFP ratio in cells with pbzpR-17 was not due to differences in transcription as RT-PCR showed that the levels of RFP and GFP mRNAs were essentially the same within these cells and also the same as those in control cells possessing pbzpR-14 (Fig. 6C).

To examine if the translational suppression was due to the uORFs within the *bzpR* 5′ UTR, the start codon of each of the 7 uORFs was destroyed individually (ATG to AAG). In each case, similar suppression was seen to that of the unmutated 5′ UTR. A number of pair wise combinations of start site mutations were also examined and similarly found not to relieve the suppression. We thus made a more drastic alteration in the 5′ UTR by deleting the internal 5 uORFs and fusing the beginning of the first and end of the seventh uORFs to give a 5′ UTR with a single uORF (pbzpR-53; Fig. 1). Surprisingly, the single 5′ UTR resulted in greater translational suppression, with essentially no GFP protein being produced (Fig. 6A and 6B). Importantly, the suppression was relieved when the start codon of the single uORF was mutated (pbzpR-54), indicating the uORF was responsible for repressed translation. RT-PCR showed a small reduction in GFP mRNA in pbzpR-53 cells relative to pbzpR-54 (Fig. 6C). However, the 96 fold increase in GFP fluorescence in pbzpR-54 cells indicates that loss of translation suppression upon mutating the start site of the uORF accounts for the vast majority of increased GFP production in pbzpR-54 cells.

## Discussion

In response to a variety of stresses, phosphorylation of eIF2α results in the enhanced translation of mRNAs of the bZIP transcription factors GCN2 in yeast and ATF4 and ATF5 in mammals to generate changes within the stressed cells to cope with the particular stress [3], [5], [7], [10], [11]. The results presented herein demonstrate that production during the developmental program of a *Dictyostelium* bZIP transcription factor, BzpR, is regulated by eIF2α phosphorylation by the IfkA and IfkB kinases. While the mammalian eIF2α kinase PERK has been shown to be required for proper mouse development, with PERK null mice showing defects in skeletal and pancreas development, downstream effectors involved in developmental regulation by PERK have not been identified [25]. Hence, to our knowledge this is the first demonstration of translational control mediated by eIF2α phosphorylation and an identified downstream translational target that functions to regulate aspects of development. While the developmental defects remain to be examined in detail, strains lacking BzpR, either due to being null in the *bzpR* gene or because of an inability to produce BzpR due to the S51A mutation in eIF2α, result in very small developing structures and produce small fruits with weak and defective stalks and nonviable spores.

Fusion of the *bzpR* promoter and 5′ UTR to the *lacZ* gene resulted in constitutively produced mRNA but spatially and temporally regulated production of β-galactosidase protein. In wild type cells, protein production was observed in fingers, slugs, and early culminants in a pattern suggestive of expression in anterior like cells (ALCs) within the prespore region [26]. A speckled pattern was also seen in early finger/slugs within the anterior or prestalk region, but expression in the prestalk region was lost in late finger/slugs and early culminants. In mid to late culminants, a prominent band of intensely stained cells was observed in the upper cup region. Upper cup cells are derived from ALCs as are lower cup and basal disk cells [27], [28]. The lack of β-galactosidase in the latter two cell types suggest its production was confined to the recently identified PstU cells [29]. Normal production of BzpR in upper cup or PstU cells may explain the “droopy” sori phenotype of *bzpR* null cells, as upper cup cells have been shown to function in lifting the spore mass up the stalk [30].

Little to no production of β-galactosidase protein from mRNA containing the *bzpR* 5′ UTR was seen in the BS167 strain, i.e., in cells that possess an eIF2α that cannot be phosphorylated. Lack of protein production was found even though levels of *lacZ* mRNA containing the *bzpR* 5′ UTR were comparable in BS167 and wild type cells, and the levels were unchanging during growth and development in both strains. These results indicate that protein production from mRNA containing the *bzpR* 5′ UTR was dependent on eIF2α phosphorylation. This dependency was confirmed by the finding that little to no β-galactosidase was produced in strains that lacked either of the eIF2α kinases IfkA or IfkB. Significantly, IfkA and IfkB are expressed in ALCs in fingers and slugs, and in culminants, IfkA is expressed only in upper cup cells while IfkB is expressed in both upper and lower cup cells [13]. Thus, the localization of these kinases corresponds well to the normal spatial pattern of protein production from mRNA containing the *bzpR* 5′ UTR.

Fusing the *bzpR* 5′ UTR and first nine codons to the GFP gene resulted in severe suppression (12 fold) of translation of the GFP protein when compared to a GFP gene fused only to the ribosome binding site and first 9 codons (no-UTR). The *bzpR* 5′ UTR is complicated by having 7 uORFs. The prototypical UTR regulated by eIF2α phosphorylation is the GCN4 gene, which has four uORFs [3], [9], and another well characterized UTR is that of mammalian ATF4 which uses two uORFs [5], [31], [32]. Under conditions of low eIF2α phosphorylation, reinitiation by a ribosome at a downstream uORF following translation of the first uORF leads to release of the ribosome after translation at the reinitiated site, and thus the scanning ribosome fails to reach the true coding region [1]. Phosphorylation of eIF2α results in delayed reinitiation such that the scanning ribosome reinitiates mostly at the true coding region instead of at an uORF.

A mutation destroying any one of the uORF start codons and pair wise combinations of these mutations did not relieve the repression of translation by the *bzpR* 5′ UTR. Deleting uORFs 2 through 6 and fusing the first and last uORF to give a 5′ UTR with a single uORF resulted in even greater suppression of translation than the intact 5′ UTR, with little to no GFP being detected. However, when the start site of this single uORF was destroyed, translation was increased by more than 90 fold. Comparison of typical *Dictyostelium* start codon contexts [33] with those of the *bzpR* uORFs indicates that uORF 2 and 3 possess a somewhat rare context while the sequence contexts of 6 and 7 are quite poor. Thus, it is unclear if all seven of the uORFs are translated by ribosomes.

While the *bzpR* 5′ UTR has 7 uORFs, a single uORF is clearly sufficient for repression. Recent work with CHOP mRNA also demonstrated translational repression by eIF2α phosphorylation through a single uORF [10]. The differences in levels of repression between the single uORF 5′ UTR and the intact *bzpR* 5′ UTR suggest that the various uORFs contribute in subtle ways to a variable repression by the *bzp* 5′ UTR. It may be that the 7 uORFs allow variation in the levels of de-repression in the presence of varying amounts of phosphorylated eIF2α. A more systematic mutational analysis will be required to tease out the mechanistic subtleties of the 7 uORFs.
